# Pineal Parenchymal Tumors of Intermediate Differentiation: A Case Report and Literature Review

**DOI:** 10.7759/cureus.50139

**Published:** 2023-12-07

**Authors:** Alaa M Samkari, Fayez D Alshehri, Abeer S AlMehdar, Mutaz Y Matar

**Affiliations:** 1 Department of Molecular and Neuropathology, Department of Pathology and Laboratory Medicine, King Abdulaziz Medical City Ministry of National Guard Health Affairs, Jeddah, SAU; 2 Department of Neurosurgery, King Abdulaziz Medical City Ministry of National Guard Health Affairs, Jeddah, SAU; 3 Department of Radiology, King Abdulaziz Medical City Ministry of National Guard Health Affairs, Jeddah, SAU; 4 Department of Neurosurgery, Al Noor Specialist Hospital, Makkah, SAU

**Keywords:** pptid, management strategies, adjuvant therapy, surgical resection, pineal gland tumors

## Abstract

Pineal parenchymal tumors of intermediate differentiation (PPTIDs) account for a significant proportion of pineal tumors and are classified as grade II/III according to the WHO classification. The management of PPTIDs remains controversial because of limited reported cases and the absence of standardized treatment guidelines. We present a case of an eight-year-old male child who presented with vomiting and a sudden squint of the eyes. Imaging revealed a well-defined heterogeneous enhancing lesion in the pineal region with acute hydrocephalus. The patient underwent surgical resection, and the tumor was diagnosed as PPTID. Local recurrence occurred 10 months later, requiring a second surgical intervention and adjuvant radiation therapy. A follow-up showed a regression of the tumor and improvement in symptoms. A literature review of reported PPTID cases revealed variability in clinical presentation, treatment approaches, and outcomes. Headaches were the most common symptom, and surgical resection was the primary treatment modality. Adjuvant therapies such as radiation therapy and chemotherapy were utilized in some cases. Tumor recurrence was observed in several instances, underscoring the need for long-term follow-up. In conclusion, PPTIDs are rare brain tumors with challenging diagnosis and management. Surgical resection remains the mainstay of treatment; however, the optimal approach is uncertain. Standardized reporting and larger studies are necessary to establish guidelines for the management of PPTIDs and improve long-term outcomes.

## Introduction

Pineal gland tumors are rare brain tumors. These account for about 1% of all types of intracranial tumors [1]. These tumors can be classified histologically based on their tissue origin: germ cell tumors, pineal parenchymal tumors, tumors of supportive structures, nonneoplastic, and metastatic [2]. Of all these pineal tumors, pineal parenchymal tumors account for 14%-27% of the reported cases in the literature [3]. According to the WHO classification of pineal gland tumors, pineal parenchymal tumor of intermediate differentiation (PPTID) is classified as grade II/III [2]. PPTIDs account for about 20% of the pineal parenchymal tumors found in young adults and children [2]. Moreover, PPTIDs are divided morphologically into three subtypes: (1) lobulated endocrine-like with high vascularity; (2) diffuse growth patterns, like oligodendroglioma/neurocytoma; and (3) transitional type with areas of lobulated and diffuse growth patterns, associated with areas of pineocytomatous rosettes [4-6]. Radiologically, PPTID is difficult to distinguish from other types of pineal tumors as there are no pathognomonic features in neuroimaging [7]. There is no generally accepted overall management for these tumors [7]. Most cases require a CSF diversion procedure as many of these patients will present clinically with obstructive hydrocephalus [4]. Surgical resection +/- radiation therapy is the acceptable treatment modality [3,4]. However, because of the limited cases reported in the literature, long-term outcomes and treatment options remain controversial [3]. This paper aims to report a rare case of pineal parenchymal tumor of intermediate differentiation and to review all the cases reported in the literature.

## Case presentation

**Figure 1:**
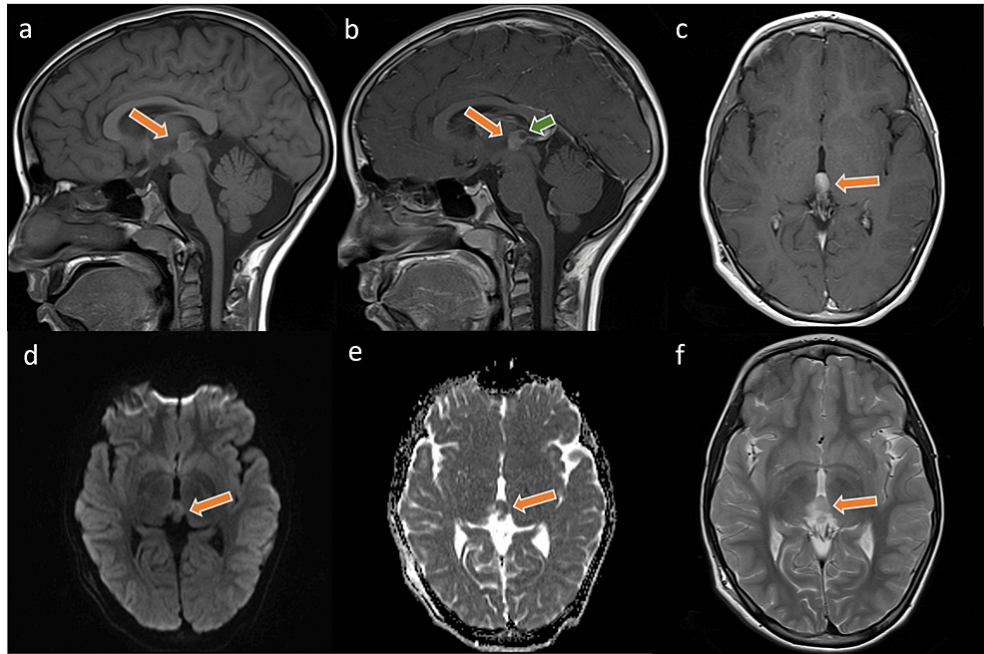
MRI images at initial diagnosis Few selected MR images of the brain at the time of the initial diagnosis demonstrate the well-defined pineal lesion (arrow). The lesion is of an intermediate signal in the T1-weighted images with a rim of high signal (a) and low T2-weighted images signal intensity (c) with enhancement on the post-contrast images as seen in the sagittal (b) and axial (f) planes. Noted is the diffusion restriction signal in the DWI sequences (d, e). Intralesional cystic (green arrow) changes are identified and no evidence of tectal infiltration was found.

An eight-year-old male child who was previously healthy presented to a local hospital with a history of vomiting and sudden squint of his eyes. The patient was diagnosed with a lesion in the tectal area and acute hydrocephalus and a ventriculoperitoneal shunt (VP shunt) was inserted at that time. An MRI of the brain showed a well-defined heterogeneous enhancing lesion centered in the pineal region with multiple small cystic components. The lesion was predominantly low in T1- and T2-weighted images and measures about 1.5 x 1.6 x 1.9 cm with extension to the posterior third ventricle and with no evidence of spinal metastasis (Figure 1).

**Figure 2:**
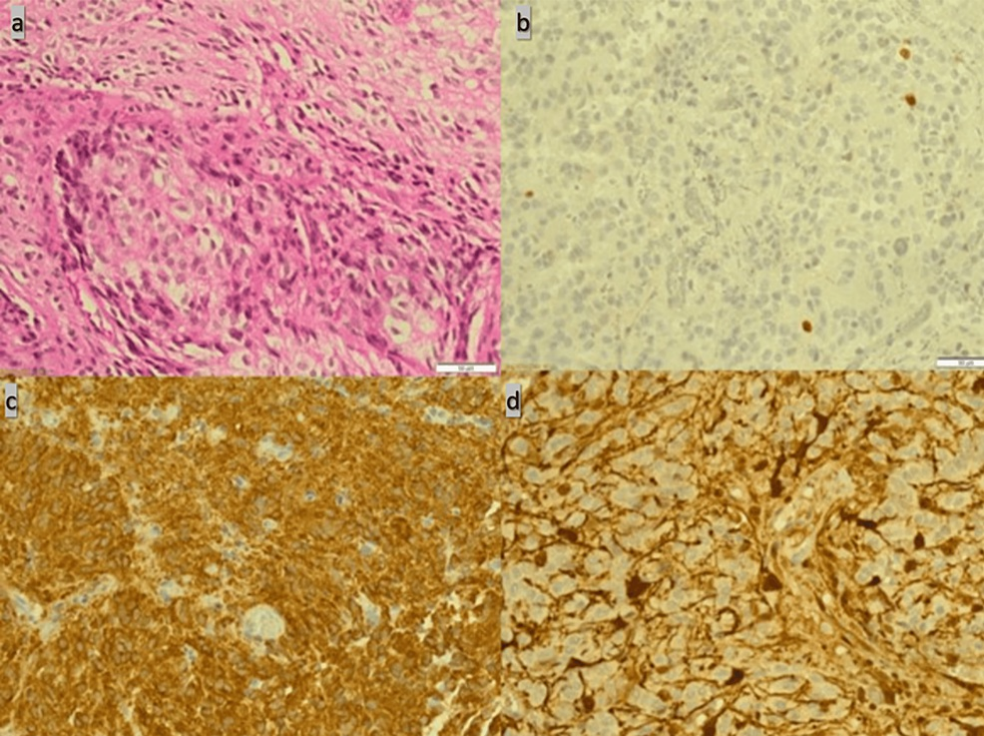
Histopathological description Pathology slides show monomorphic, round/oval nuclei with occasional perinuclear clearing and microcalcification (a). Ki-67 immunolabeling reaches up to 5% (b). Additionally, there is a focal glial fibrillary acidic protein (GFAP) and synaptophysin immunostaining (c and d).

The patient was admitted and underwent a near-total surgical resection of the tumor via a suboccipital approach. Postoperative recovery was uneventful, and a sample of the tumor was sent for pathological analysis. A microscopic examination of the tumor cells showed monomorphic, round/oval nuclei with occasional perinuclear clearing and microcalcification. Tumor cells were immunopositive for CK8/18, S100, and immunonegative for epithelial membrane antigen (EMA). Additionally, there was focal glial fibrillary acidic protein (GFAP) and synaptophysin immunostaining. Ki-67 immunolabeling was estimated to reach 5%. The tumor was diagnosed as a pineal parenchymal tumor of intermediate differentiation with a WHO grade II (Figure 2).

**Figure 3:**
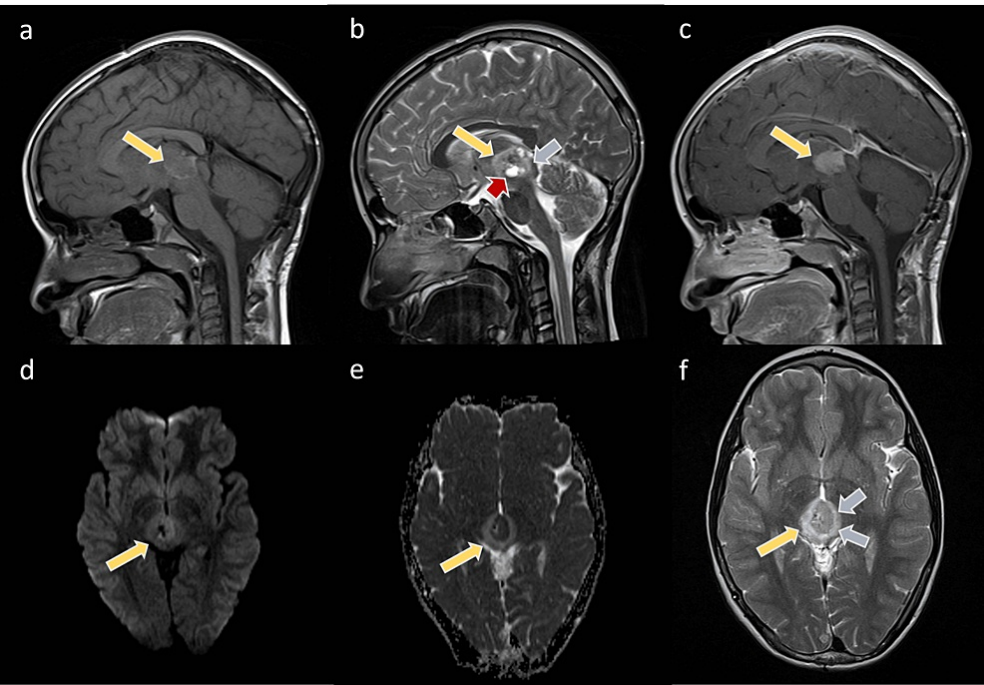
Selected MRIs of the patient after the recurrence of the mass The images show the pineal mass larger than the initial lesion (yellow arrows). The mass shows low to intermediate T1 signal intensity with the bright surrounding rim (a) and heterogeneous low T2 signal intensity (b, f)) with intralesional cysts (red arrow). The mass is enhanced in the post-contrast T1WI sequence (c) and restricted on the DWI (d,e). Noted is the surrounding edema (blue arrows) of the bilateral thalami and tectum on the T2WI.

Ten months later, an MRI of the brain showed local recurrence (Figure 3), and a second surgical intervention was done via a suboccipital midline approach. Postoperatively, the patient was stable and shifted to the PICU. A postoperative examination of the patient showed vertical gaze palsy, most likely dorsal midbrain (Parinaud's) syndrome. An adjuvant focal radiation therapy was given, and regression of the tumor was noted on follow-up images.

**Figure 4:**
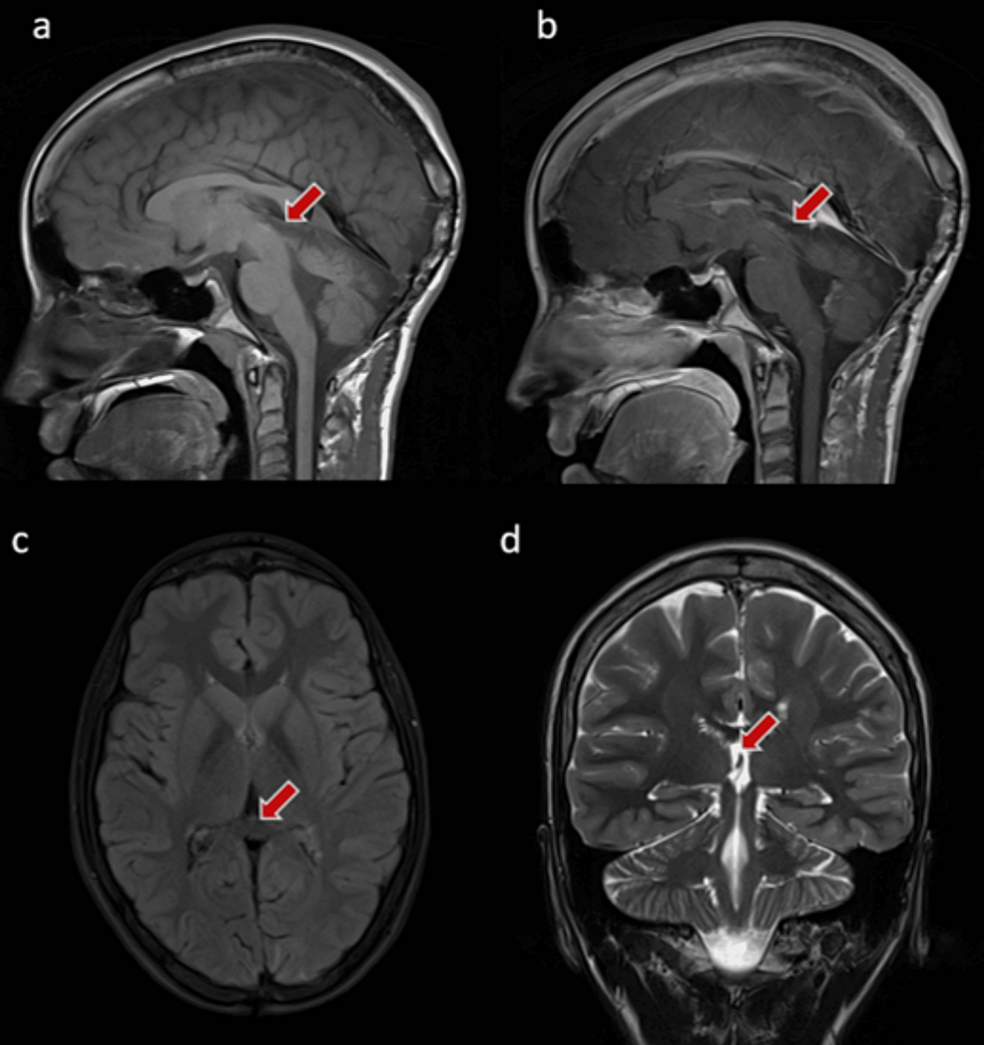
Selected images of the brain MR after the resection (two years follow-up) The images show the clear pineal area with no evidence of residuals or masses (red arrow).

At a nine-month follow-up after completing radiation therapy, the patient was doing fine with an improvement of the upward gaze palsy. Follow-up imaging showed a further decrease in tumor size with no evidence of metastasis to other parts of the body (Figure 4).

## Discussion

Pineal parenchymal tumors are extremely rare brain tumors and account for approximately 1% of all adult primary intracranial malignancies [8]. They encompass different types of tumors, including well-differentiated pineocytoma, poorly differentiated pineoblastoma (PB), and PPTIDs. PPTIDs comprise a significant percentage (21% to 54%) of all pineal parenchymal tumors reported in various studies, but the variability in reported incidence is attributed to challenges in diagnosing the spectrum of pineal parenchymal tumors [9,10].

The literature review includes a total of 72 reported cases of PPTIDs. The mean age of the reported cases is 33.6 years, with a range of 2-75 years. The gender distribution among the reported cases is relatively balanced, with a male-to-female ratio of approximately 1:1. The most common clinical presentation was headaches. Focal neurological signs were observed in some cases, including diplopia, ataxia/vertigo, lower-extremity weakness, gait disturbance, and Parinaud's syndrome in addition to lethargy, blurry vision, confusion, and limb weakness [11,12].

The specific tumor size is not consistently mentioned in the literature. However, the reported cases include tumor sizes ranging from 2.0 to 7.6 cm, with a mean size of 3.1 cm. Primary treatment approaches vary, with surgical resection being the most common. Adjuvant therapy, such as radiation therapy (XRT), chemotherapy (CTx), or craniospinal irradiation (CSI), is reported in some cases.

Outcomes vary among the reported cases. Tumor recurrence was noted in several instances, with recurrence reported in 7 out of 17 cases in the study by Nam et al. The follow-up period ranges from 0.1 months to 21 years, with a mean follow-up of 45.3 months. Other studies also report cases of tumor recurrence, while some cases remain tumor-free after treatment [13,14].

Comparing the reported cases, it is evident that there is variability in the clinical presentation, treatment approaches, and outcomes. The lack of consistent reporting of tumor size and treatment details limits the ability to draw definitive conclusions about the most effective management strategies for this condition. Further studies with larger sample sizes and standardized reporting are necessary to obtain a better understanding of the clinical characteristics, optimal treatment approaches, and long-term outcomes for patients with this condition [15-18]. Table 1 shows the literature review of the reported cases.

**Table 1:** Reported PPTID cases in the literature Y = years, M = Male, F = Female, Pt = Patient, GTR = Gross Total Resection, PTR = Partial Tumor Resection, STR = Subtotal Resection, XRT = Radiation Therapy, CTx = Chemotherapy, CSI = Craniospinal Irradiation, SRS = Stereotactic Radiosurgery, cm = Centimeter

No	Case report	Age and gender	Clinical presentation	Tumor size	Primary treatment	Surgical approach	Adjuvant therapy	Outcome	Follow-up period
1	Pusztaszeri 2006 [2]	28 Y, F	Progressive, atypical headaches	3.2 × 1.2 × 1 cm	CTx + CSI	-	-	At six years, no evidence of recurrence	Six years
2	Anan et al. 2006 [3]	60 Y, M	Memory disturbance, gait instability, and double vision	Not mentioned	PTR	Right occipital transtentorial approach	XRT+CTx+ SRS	Decreased tumor size	Two years
3	Senft et al. 2008 [4]	44Y, M	Flash-like visual impairment, headache, and gait disturbances	2.2 cm x 2.2 cm	STR	Trans-ventricular/trans-choroidal fissure approach	SRS	No evidence of tumor Recurrence	One year
4	Shimada et al. 2008 [5]	12 Y, F	Headache and vomiting and obstructive hydrocephalus	Not mentioned	SRS	Not mentioned	PTR	Not mentioned	Not mentioned
5	Kim et al. 2009 [6]	47 Y, F	Asymptomatic, incidental finding	3.2 cm	Biopsy only	ETV + biopsy	SRS + surgical resection + CTx	Recurrence after four years	Four years
6	Komakula et al. 2010 [7], 11 cases reported	Mean 23 years (4.5–75) M:F ratio: 7:4	Headache (8/11), Parinaud syndrome (3/11), gait disturbances (3/11), other unspecified visual symptoms (2/11), and seizures (1/11)	Mean: 2.5 cm (1-6 cm)	PTR: four others not mentioned	Not mentioned	XRT+ CTx: three cases, XRT only: three cases	Recurrence: three cases	Six months–21 years
7	Li et al. 2010 [8]	14 Y, F	Headaches	3.6 cm	GTR	Infratentorial, supra-cerebellar approach	Not mentioned	Not mentioned	Not mentioned
8	Fukuoka et al. 2012 [9]	11 Y, F	Progressive headaches and nausea	4.8 cm	PTR	Occipital transtentorial approach	XRT + CTx + SRS	No recurrence after 17 months	17 months
9	Ito et al. 2014 [10], six cases	Mean age of 62.3 years (range: 32-71) years 1 M, 5 F	Not mentioned	Not mentioned	PTR: 3, biopsy only: 2, STR: 1	Not mentioned	XRT + CTx: three cases, XRT only: two cases	Recurrence: three cases, no recurrence: three cases	Three months–three years
10	Patil et al. 2015 [11]	25 Y, F	Headache, nausea, vomiting, eye discharge	Not mentioned	Not mentioned	Not mentioned	Not mentioned	At three years, the patient presented with metastasis	Three years
11	Yu et al. 2016 [12], 27 cases	29.7 Y (range: 2-62), M:F ratio: 1:1	Headache (18 cases), nausea and vomiting (13 cases), impaired vision (three cases), gait disturbance (four cases), diplopia (four cases), hearing impairment (one case), and limb weakness (three cases)	2.0 cm to 6.0 cm (mean, 3.1 cm)	GTR in 16 patients (59.3%), STR in six patients (22.2%), PTR in five patients (18.5%)	Transcallosal 15 (55%), occipital transtentorial (33%), transcortical 3(11%)	SRS: two (7.4%), XRT: 17 (63%), None: eight (29.6%)	Recurrence or progression: seven (25.9%), mortality: five (18.5%)	Follow-up time ranged from 18 to 107 months (mean, 45.3 months)
12	Kang et al. 2016 [13]	24 Y, M	Headaches, gait abnormalities, and abulia; anisocoria with left-sided mydriasis; restricted up gaze; left pronator drift; dysmetria; intention tremor; hypophonia; limited speech output	7.6 cm x 6.8 cm	GTR	Occipital transtentorial and suboccipital infratentorial supra-cerebellar approaches	CSI+ CTx	Ambulating independently and conversant with caretakers, but continued to exhibit psychosocial delay	Four months
13	Yoon et al. 2016 [14]	25 Y, F	Lethargy, blurry vision, confusion and headache	2.2 cm	GTR	Suboccipital, supra-cerebellar approach	None	No residual tumor, patient's symptoms improved	One year follow-up
14	Bando et al. 2018 [15]	63 Y, F	Bilateral lower-extremity weakness and gait disturbance, Parinaud’s syndrome	Not mentioned	GTR	Right occipital transtentorial	SRS + CSI + CTx	The patient had tumor recurrence at six months, and two years. At four years, no evidence of recurrence	Four years
15	Nam et al. 2020 [16], 17 reported cases	Median age 37 Y (15-57), M:F ratio: 1:1	16/17 patients headaches, focal neurological signs; for 5/17, diplopia was the most common symptom; and for 2/17 presented with ataxia/vertigo	Not mentioned	Biopsy: 3, GTR: 7, STR: 6	Not mentioned	XRT: 16, XRT+ CSI: 12, CTx: 4	Seven had tumor recurrence	62.6 months (range, 0.1-162.8 months)

## Conclusions

PPTIDs are rare brain tumors that account for a significant percentage of pineal parenchymal tumors. The diagnosis of PPTIDs is challenging because of the lack of pathognomonic radiological features, and histopathological analysis is necessary for a definitive diagnosis. Surgical resection is the primary treatment modality, often accompanied by adjuvant therapies such as radiation therapy. However, optimal management strategies for PPTIDs remain controversial because of limited reported cases and the lack of standardized treatment approaches. A literature review of reported cases revealed variability in clinical presentation, treatment approaches, and outcomes. Larger studies with standardized reporting are needed to establish guidelines for the management of PPTIDs and improve long-term outcomes for patients.
